# Editorial Correspondence from Warm Springs, N. C.

**Published:** 1875-10

**Authors:** W. F. Westmoreland

**Affiliations:** Warm Springs, N. C.


Warm Springs, N. C., August 17th, 1875.

*Dear Journal:*

In my letter of the 11th instant, I promised in my next to discuss the medicinal qualities of the waters at this place, and the class of diseases likely to be benefited by them. As the warm bath is the prominent feature here, a few remarks in the way of elucidating the following propositions, in regard to the therapeutic action of thermal waters, will be necessary to a proper understanding of my opinion as to their value.

1st. What is the difference between the heat of thermal waters and heat artificially induced?

2d. If there is no therapeutic difference in artificial and natural heat, what advantage, if any, has the natural warm bath over the artificial warm bath?

3d. What is the difference in the physiological effects of both at different temperatures?

4th. To what extent are the mineral ingredients in solution, or in the form of gases in thermal waters absorbed, and at what temperature is absorption most active?

I feel that a correct solution of the above problems is essential to a proper estimate of the therapeutic value of all thermal baths. Up to a recent date the opinion almost universially prevailed that there was a marked difference between the therapeutic effect of natural and artificial heat, many contending for the thermo-electric properties of thermal waters. More recent investigations, however, have decided that there is no difference. In a letter upon this subject, to Dr. George E. Walton, of Cincinnati, Prof. Tyndall, of London, who is certainly the very highest authority on heat, says: "I am not acquainted with any difference between natural and artificial heat. I am not acquainted with any thermo-electric condition that could cause any perceptiable difference between the therapeutic action of natural hot water and artificial hot water."

Admitting, as we must then, that there is no difference, still the fact remains that natural hot or warm baths are much more efficient and active medicinal agents than artificial hot or warm baths. This difference may be accounted for to some extent by the absorption of mineral ingredients, either in the gaseous form or held in solution by the water; but, in my opinion, mainly by the uniform and unchanging temperature of the thermal baths. An artificial hot or warm bath is never uniform, but the temperature is constantly changing from the time the water enters the bathing-tub, until the patient leaves it, while the large pools at the hot or warm springs are ever the same temperature.

To better understand their physiological effects, thermal baths have been divided into tepid, warm and hot. A bath at from 85° to 90° Fahr. is termed a tepid bath; from 92° to 98°, a warm bath, and all above 98° are termed hot baths. All baths with a temperature below animal heat are sedative, while all above animal heat are exciting—thus a warm bath at a temperature from 92° to 98° Fahr. will decrease the frequency of the pulse, and the number of respirations per minute, while a bath much above 98° will increase the frequency of the pulse and the number of respirations per minute.

Dr. Lockett, of Virginia, who has thoroughly investigated this subject, found that in a bath of 98° Fahr. his pulse was 77 to the minute, but in a bath of 111° Fahr. it rose to 153 beats in the minute, producing a train of nervous symptoms, such as dimness of sight, *tinitus aurium*, etc.

The extent to which mineral ingredients held in solution, or in a gaseous form are absorbed, are often greatly over estimated, as is so frequently demonstrated at localities where so much is claimed, from the absorption of the mineral ingredients, but where the baths are used at such an elevated temperature as to make absorption to any extent impossible. Under favorable circumstances, however, absorption does take place, and in sufficient quanties to be very perceptible. It has been often demontrated that absorption is most active in baths of a temperature from 86° to 96° Fahr., and decreases as the temperature of the bath is increased, until at a temperature of 110° to 120° Fahr. it is hardly perceptible.

*En resuma*, we find, then, that while there is no difference between artificial heat and natural heat, there is a marked difference in the therapeutic action of a natural warm or hot bath and an artificial warm or hot bath.

That the physiological effects of thermal baths differ very greatly—that a bath the temperature of which is much above animal heat (98°) will excite the functions of the vital organs as the brain, heart, and lungs, while a bath the temperature of which is below animal heat acts as a sedative, reducing the force and frequency of the heart's action, the number of respirations per minute, etc.

That the amount of mineral ingredients absorbed is often greatly over estimated. That baths at a temperature of from 86° to 96° Fah. is most favorable to absorption.

The Warm Springs are situated in Madison county, North Carolina, the extreme northwestern portion of the State, on the beautiful French Broad River, and in the loveliest valley in the world, from which arise ten thousand peaks of mountains, which seem to the eye to cover all space. The scenery is enchanting, the air pure and bracing, and in fact the surroundings are all that could be desired in a health-giving point of view. We have here several Warm Springs, chalybeate and sulphur springs, [cold,] and the very best freestone water—the latter brought from a mountain, on the opposite side of the river, in iron pipes. The

Warm Springs are the greatest feature here. As but little has been done in the way of developing the chalybeate and sulphur springs, this being the first season they have been used, no analysis has as yet been made of them. However, from the taste and surroundings, I would judge the sulphur spring to be strongly impregnated with sulphur and iron, with perhaps other minerals. For the analysis, chemical properties, temperature, surroundings, etc., I cannot do better than give the following page from that most valuable little book, "The Mineral Springs of the United States and Canada," by George E. Walton, M.D., of Cincinnati:

ANALYSIS OF WARM SPRINGS, N. C.

| One pint contains— | Bathing Springs. 102° Fahr. E. Adelmarth, M. D. | Drinking Springs. 97° Fahr. E. Adelmarth, M. D. |
|---|---|---|
| SOLIDS. | Grains. | Grains. |
| Chloride of potassium | 0.039 | 0.063 |
| Chloride of sodium | 0.114 | 0.137 |
| Chloride of magnesium | 0.027 | 0.046 |
| Chloride of calcium | 1.263 | 1.118 |
| Sulphate of potassa | 0.045 | 0.059 |
| Sulphate of soda | 1 128 | 1.113 |
| Sulphate of magnesia | 0.168 | 1.016 |
| Sulphate of lime | 5.110 | 5.067 |
| Soluble silicates | 1.121 | 1.192 |
| Total | 9.015 | 9.711 |
| GASES. | Cubic in. | Cubic in. |
| Carbonic acid | 1.37 | 1.34 |
| Sulphuretted hydrogen | 0.22 | 0.21 |

*Properties.*—Chemically considered, these are calcic-sulphur waters, bearing considerable resemblance to the well-known baths of Leuk, in the valley of the Rhone, Switzerland. They are valuable thermal waters, and are efficacious in *chronic rheumatism, gout, paralysis, dartrous skin-diseases*, and irritable conditions of the urinary apparatus. They also are useful in certain cases of *amenorrhœa* and *dysmenorrhœa.*

*Remarks.*—The Warm Springs are at an elevation of thirteen hundred and twenty-five feet above the sea, surrounded on all sides by pine-covered mountain-summits, save the gorge and valley where the French Broad River has worn its pathway. The hotel and cottages are included in an erea of about one hundred and fifty acres, well shaded, and interwoven with winding walks. The scenery of the region is exceedingly wild and beautiful. The banks of the river are precipitous in many places, at the springs being over one hundred feet in height. At a distance of one-fourth of a mile from the hotel is Lover's Leap, an elevated point, frequently visited by tourists, whence a far-extended view, for many miles, is had of the winding and turbulent river and the enclosing mountain-peaks. The climate is cool and bracing, the severe heats of summer being unknown.

"The springs are near the banks of the river. One, the largest, is enclosed by a brick-wall laid in cement, and has a bath-house built over it. The bath is divided into two compartments—one for ladies, the other for gentlemen. The swimming-baths are about twenty by thirty feet, and four and a half feet deep. The mean temperature of the ladies' bath is 102° Fahr.; the gentlemen's, 100° Fahr. The flow of water is constant, averaging nine gallons per minute."

It will be seen that the above analysis of the two springs differ slightly in their mineral constituents and in temperature; one is used for drinking, and has a considerable reputation as an anti-dyspeptic, being slightly cathartic and diuretic when taken in large quantity; the other is used exclusively for bathing purposes. A striking peculiarity of this locality is, that a spring may be had at almost any point for the digging. I mean that at numerous points, up and down the river, a shaft sunk to the depth of from two to six feet will develop a hot spring at from 95° to 107° Fahr. Two days ago, to demonstrate this peculiarity, Mr. Rombough, the proprietor, had a shaft sunk three feet deep and as many in diameter, on what is called "the island," two or three hundred yards from the hotel, and in a very short time it was filled to the surface with water at a temperature of 106½° Fahr., several degrees, as will be seen, warmer than the spring now in use. Of one fact there can be no doubt, that any quantity of water at from 95° to 107° Fahr. can be found at various points for a mile or more up and down the river from the hotel, with an analysis similar to the one above given.

How do the baths act, is the question so often asked and so differently answered. Are the great benefits derived the result of the absorption of one, or more, or all the mineral ingredients? or are the results attributable to the elevated temperature of the bath? To the latter, I feel confident, is due very nearly if not all therapeutic action of the baths here as well as all thermal baths.

We have seen that baths over 98° Fahr. do not favor absorption, and as we have here a temperature of 102° Fahr., but little absorption can take place. Admitting that all the ingredients in solution were absorbed, and in the quantities desired, we could not then account for the effects we see here daily. At the Hot Springs in Arkansas, where so much is claimed by the uneducated, and I am sorry to say by some scientific gentlemen, as the result of the absorption of the mineral ingredients contained in the water, it is evident that none of the effects claimed for the baths is due to absorption, but simply to the elevated temperature. Dr. George E. Walton, who has given this whole subject more thought than any professional man in this country, in answering the question as to how the waters at the Hot Springs act, says: "Principally, if not entirely, by elevated temperature."

The diseases which are likely to derive most benefit from the baths here are rheumatism, gout, paralysis unaccompanied by organic lesions, dartrous skin affections, irritable conditions of bladder and urinary organs, dysmenorrhœa, amenorrhœa, and as an auxiliary syphilis in its varied forms as syphilitic rheumatism, syphilitic periostitis, etc., etc. Stiff joints, from either injuries or specific disease, with the proper appliances, would be benefitted.

While I have not seen the effects of the baths in all the diseases above mentioned, I have seen enough to convince me that all would be more or less benefitted, and some cured, by their judicious use. I have seen one case of dysmenorrhœa, rheumatic in character, that had for years resisted all medecinal agents, yield promptly to the use of the baths. I have met here several rheumatics who assure me that they have been greatly benefitted by their use. Some who have visited the Hot Springs in Arkansas tell me that they derive as much benfit from the baths here as at the Hot Springs, and I believe them, as I can see no reason why, with the same care and attention, the baths here will not accomplish the same results as the baths at Hot Springs. The only difference in the waters that amounts to much is the temperature; but as the temperature can be had, as above stated, at $106\frac{1}{2}^{\circ}$ Fahr.,—hot enough for all practical purposes—I can't for my life see why the baths here could not be made to accomplish the same results as those claimed for Hot Springs. As I am confident this statement will be questioned by many, I will here give the analysis of the Hot Springs of Arkansas from the valuable little book of Dr. Walton, above quoted:

ANALYSIS OF HOT SPRINGS, ARKANSAS.

One Pint contains (93°–150° Fahr.):

| Solids. | Grains. |
|---|---|
| Carbonate of magnesia | 0.016 |
| Carbonate of lime | 0.496 |
| Chloride of sodium | 0.001 |
| Sulphate of potassa | 0.029 |
| Sulphate of soda | 0.047 |
| Sulphate of lime | 0.014 |
| Sesquioxide of iron | 0.013 |
| Iodine | trace. |
| Bromine | trace. |
| Silicate of lime, | 0.058 |
| Silica | 0.233 |
| Alumina | 0.056 |
| Organic matter | 0.088 |
| Water | 0.018 |
| Total | 1.069 |

It will be seen that the quantity of mineral ingredients, if they be of value, is much greater here than at Hot Springs, there being no great difference in the chemical properties of the two. The greater range of temperature of the Hot Springs constitutes the only important difference.

The trouble here is that the baths are not sufficiently numerous for the number of bathers, nor are they arranged and fitted up with appliances that are essential to the most favorable results in the treatment of some affections. Upon this point, however, I am assured by the proprietor that everything will be in readiness by the opening of the next season. Another advantage that Hot Springs has over this place is, that at Hot Springs there is always present a corps of scientific physicians, who thoroughly understand the effects of the baths, and the invalids visiting the place at once place themselves under the direction of a physician, who administers such remedies as may be indicated in addition to directing the course of bathing, and the patient is rapidly relieved—the baths, in a number of instances, acting only auxiliary in the treatment.

In syphilitic affections, and particularly syphilitic rheumatism and periostitis, a class of affections in which the Hot Springs has made its greatest character, the remedies of the physician have done, as much or more than the baths.

I would not be understood, in what I have said, as trying to disparage the Hot Springs, for I truly regard them as of the greatest value, but my object is simply to show what is necessary here to develope and make prominent the valuable baths of the Warm Springs.

But I must close, as I have made this letter much longer than I intended, and still have not said all I desired. At some future time I hope to make another visit to this lovely retreat, and you will hear from me again. I learn to-day that Col. Rombough will likely dispose af a large interest in this valuable property, that the springs and the surroundings may be put in the most perfect order, by the opening of the next season.

Very truly, W. F. Westmoreland.

